# Identifying genetic variants and pathways associated with extreme levels of fetal hemoglobin in sickle cell disease in Tanzania

**DOI:** 10.1186/s12881-020-01059-1

**Published:** 2020-06-05

**Authors:** Siana Nkya, Liberata Mwita, Josephine Mgaya, Happiness Kumburu, Marco van Zwetselaar, Stephan Menzel, Gaston Kuzamunu Mazandu, Raphael Sangeda, Emile Chimusa, Julie Makani

**Affiliations:** 1 Department of Biological Sciences, Dar es Salaam University College of Education, Dar es Salaam, Tanzania; 2 Sickle Cell Program, Department of Hematology and Blood Transfusion, Muhimbili University of Health and Allied Sciences, Dar es Salaam, Tanzania; 3 Department of Biotechnology Laboratory, Kilimanjaro Clinical Research Institute, Kilimanjaro, Tanzania; 4 Department of Molecular Hematology, King’s College of London, London, UK; 5 Department of Pathology, Division of Human Genetics, University of Cape Town, IDM, Cape Town, South Africa; 6 Department of Integrative Biomedical Sciences, Computational Biology Division, University of Cape Town, Observatory, 7925 South Africa; 7 African Institute for Mathematical Sciences, Muizenberg, Cape Town, 7945 South Africa; 8 Department of Pharmaceutical Microbiology, Muhimbili University of Health and Allied Sciences, Dar es Salaam, Tanzania

**Keywords:** Sickle cell disease, Genetic disorder, Fetal hemoglobin, Hemoglobinopathy, Tanzania

## Abstract

**Background:**

Sickle cell disease (SCD) is a blood disorder caused by a point mutation on the beta globin gene resulting in the synthesis of abnormal hemoglobin. Fetal hemoglobin (HbF) reduces disease severity, but the levels vary from one individual to another. Most research has focused on common genetic variants which differ across populations and hence do not fully account for HbF variation.

**Methods:**

We investigated rare and common genetic variants that influence HbF levels in 14 SCD patients to elucidate variants and pathways in SCD patients with extreme HbF levels (≥7.7% for high HbF) and (≤2.5% for low HbF) in Tanzania. We performed targeted next generation sequencing (Illumina_Miseq) covering exonic and other significant fetal hemoglobin-associated loci, including *BCL11A*, *MYB*, *HOXA9*, *HBB*, *HBG1*, *HBG2*, *CHD4*, *KLF1*, *MBD3*, *ZBTB7A* and *PGLYRP1*.

**Results:**

Results revealed a range of genetic variants, including bi-allelic and multi-allelic SNPs, frameshift insertions and deletions, some of which have functional importance. Notably, there were significantly more deletions in individuals with high HbF levels (11% vs 0.9%). We identified frameshift deletions in individuals with high HbF levels and frameshift insertions in individuals with low HbF. *CHD4* and *MBD3* genes, interacting in the same sub-network, were identified to have a significant number of pathogenic or non-synonymous mutations in individuals with low HbF levels, suggesting an important role of epigenetic pathways in the regulation of HbF synthesis.

**Conclusions:**

This study provides new insights in selecting essential variants and identifying potential biological pathways associated with extreme HbF levels in SCD interrogating multiple genomic variants associated with HbF in SCD.

## Background

Sickle cell disease (SCD) and thalassemia are the most common hemoglobinopathies worldwide, with 270 million carriers and 300,000 to 500,000 annual births [1]. Up to 70% of global SCD annual births occur in sub-Saharan Africa. Reports show that 50 to 80% of affected children in these countries die annually [2]. Tanzania ranks fifth worldwide regarding the number of children born with SCD, estimated at 8000–11,000 births annually. 15–20% of the population are SCD carriers (HbAS) and therefore potential parents of future babies with SCD [3, 4]. Without intervention, it is estimated that up to 50% of children with SCD will die before the age of 5 years [1]. Thus, SCD intervention at early stages of life may prevent premature deaths and reduce under-five mortality.

SCD is a monogenic condition resulting from a single mutation in the β-globin gene or hemoglobin subunit beta (*HBB*), on chromosome 11, leading to the production of an abnormal β-hemoglobin chain namely hemoglobin S (HbS). SCD is a complex hemoglobin disorder with multiple phenotypic expressions that manifest as both chronic and acute complications, affecting multiple organs. Clinical manifestations vary immensely, with some individuals being entirely asymptomatic while others suffer from severe forms of the disease. The marked phenotypic heterogeneity of SCD is due to both genetic and environmental determinants [5]. A major disease modifier is the presence of fetal hemoglobin (HbF): high HbF levels are associated with reduced morbidity and mortality [6, 7].

Hemoglobin is a tetrametric molecule composed of 2-alpha-globin and 2 gamma globin molecules in HbF and 2 alpha-globin and two 2 beta-globin molecules in HbA [8]. HbF is normally expressed during the development of the fetus and starts to decline just before birth, when it is replaced by adult hemoglobin namely hemoglobin A (HbA) in normal individuals and hemoglobin S (HbS) in individuals with SCD [9]. Red blood cells of normal adults (HbAA) contain mainly hemoglobin A (HbA), with 2.5–3.5% Hemoglobin A_2_ (HbA_2_), and < 1% HbF [10]. However, 10 to 15% of adults possess higher HbF levels (up to 5.0%). Although this has no significant consequences in healthy individuals, HbF background variability in SCD can reach levels with clinical benefit to patients [11]. Consequently, efforts to understand and control the production of HbF in SCD patients may result in interventions of significant clinical benefit to individuals with SCD.

The levels of both HbF and F cells (erythrocytes with measurable amounts of HbF) are highly heritable traits [12] with up to 89% of variation being influenced by genetic factors. The remaining proportion is accounted for by age, sex and environmental factors. It is now clear that HbF is a quantitative trait which is shaped by genetic factors both linked and unlinked to the β-globin gene. Three main loci, namely *BCL11A* on chromosome 2, *HMIP* on chromosome 6, and *HBG* on chromosome 11, have been identified across populations as associated with HbF levels [13–15]. The variants in these loci have been reported to contribute 20–50% of HbF variation in non-African populations, however the impact of these variants is different from one population to another. An example is a strong variant at *HMIP*, which is rare in the Tanzanian population and hence has a smaller impact on HbF levels there [16, 17]. HbF levels in SCD, as a quantitative trait, is expected to be influenced by other polymorphisms, including insertions/deletions, rare mutations or copy number variations [15].

New genetic and proteomic techniques have led to the identification of several HbF expression regulators. *Kruppel like factor* (*KLF*1) has been reported as one of the key regulators of HbF expression with dual functions: direct activation of HbF expression through activation of β-globin [18] and an indirect silencing of γ-globin gene through *BCL11A1* [19]. Other players within the HbF regulation network that have been reported include *GATA1, FOG1* and *SOX6*, which are erythroid transcription factors and are believed to interact with *BCL11A* in HbF regulation [20]. In addition, nuclear receptors *TR2/TR4* which are associated with *corepressors of DNA methyltransferase 1* (*DNMT1*) and *lysine-specific demethylase 1* (*LSD1*) have also been implicated. *DNMT1* and *LSD1* are a part of the DRED complex, a known repressor of embryonic and fetal globin genes in adults [21]. Recently, studies of epigenetic pathways of HbF regulation have elucidated the involvement of the *nucleosome remodeling and deacetylase* (NuRD) complex [22, 23].

Despite the high prevalence of SCD in Africa, African patient populations remain understudied. Unique insight can be obtained from these patients, considering the substantial African genetic diversity and exceptional mapping resolution. The high burden of SCD in sub Saharan Africa makes it important that genetic studies, ultimately aimed at improved therapeutic intervention, are carried out in African countries. To address this, we conducted a Genome Wide Association Study (GWAS) [16, 17, 24] and candidate genotyping for HbF in Tanzanian individuals with SCD, which led to validation of known HbF variants and identification of novel ones. This report documents a follow-up study aimed at performing in-depth targeted sequencing around previously identified loci to descriptively compare, in detail, discovered polymorphisms between individuals with extreme HbF levels. For the first time, we have conducted targeted next-generation sequencing to investigate known and novel genetic variants and pathways associated with extreme HbF levels in individuals with Sickle cell disease (SCD) in Tanzania. From these selected individuals, we have identified different types of polymorphisms, including single nucleotide polymorphisms (SNPs), structural variants such as insertions and deletions (INDELS), suggesting potential modifier effects. Interestingly, key discovered variants, together with previously identified variants, are enriched in biological pathways that underlie the HbF regulation.

## Methods

### Study design and population

We performed a cross-sectional study involving the Dar-es-Salaam (Tanzania) Muhimbili National Hospital SCD cohort, which consisted of 1725 SCD patients, recruited between 2004 and 2009, for prospective surveillance, with three monthly interval visits for routine check-up [3]. These patients were subjected to folic acid (5 mg/day) and penicillin. Different hematological factors, including complete blood counts and foetal haemoglobin (HbF) quantifications, were measured during hospital visits. Written informed consent was obtained for each adult patient (> 16 years) and ethical approval given by the Muhimbili University Research and Publications Committee (MU/RP/AEC/VOLX1/33 and 2017-03-06/AEC/Vol X11/65). Informed and written consent was obtained from parents or guardians for all minor patients (≤16 years). The study involved 14 individuals confirmed to have SCD (HbSS or S-β°thalassaemia), over 5 years old, with extreme HbF levels. Excluded were individuals confirmed to be AS or AA following Hb electrophoresis and HPLC, those with HbF measured at an age of less than 5 years, with inconclusive SCD laboratory diagnosis where a repeat test for confirmation could not be performed, and individuals who were on hydroxyurea therapy.

### Phenotyping

Individuals were selected using previously collected HbF data. In this population, the median HbF was 4.6 [Interquartile range (IQR): 2.5–7.7)] [17] and therefore 0–2.5% was considered a low HbF level while 7.7% and above was considered a high HbF level.

### Sequencing

DNA was extracted from archived buffy coat samples using the Nucleon BACC II system (GE Healthcare, Little Chalfont, UK). The sequencing panel was adopted from a research panel at King’s College London and customized using Illumina DesignStudio (https://designstudio.illumina.com/). Targeted sequencing covered exons and non-coding regions around validated and candidate fetal hemoglobin-influencing loci, including *B-cell lymphoma/leukemia 11A* (*BCL11A*), *proto-oncogene, transcription factor* (*MYB*), *homeobox A9* (*HOXA9*), *hemoglobin subunit beta* (*HBB), hemoglobin subunit gamma 1* (*HBG1*), *hemoglobin subunit gamma 2* (*HBG2*), *chromodomain helicase DNA binding protein 4* (*CHD4*), *Kruppel like factor 1* (*KLF1*), *methyl-CpG binding domain protein 3* (*MBD3*), *zinc finger and BTB domain containing 7A* (*ZBTB7A*), *peptidoglycan recognition protein 1* (*PGLYRP1*) on chromosomes 2, 6, 7, 11, 12 and 19, respectively (Table 2). Selection of target regions was based on previous associated known and novel loci in the studied population and those reported recently in other populations. Sequencing was performed on the Illumina MiSeq platform at the Kilimanjaro Clinical Research Institute (*KCRI*), Tanzania, following TruSeq Custom Amplicon Low Input Kit protocol.

### Reads mapping, alignment, variant calling and variant calling quality control

**Fig. 1:**
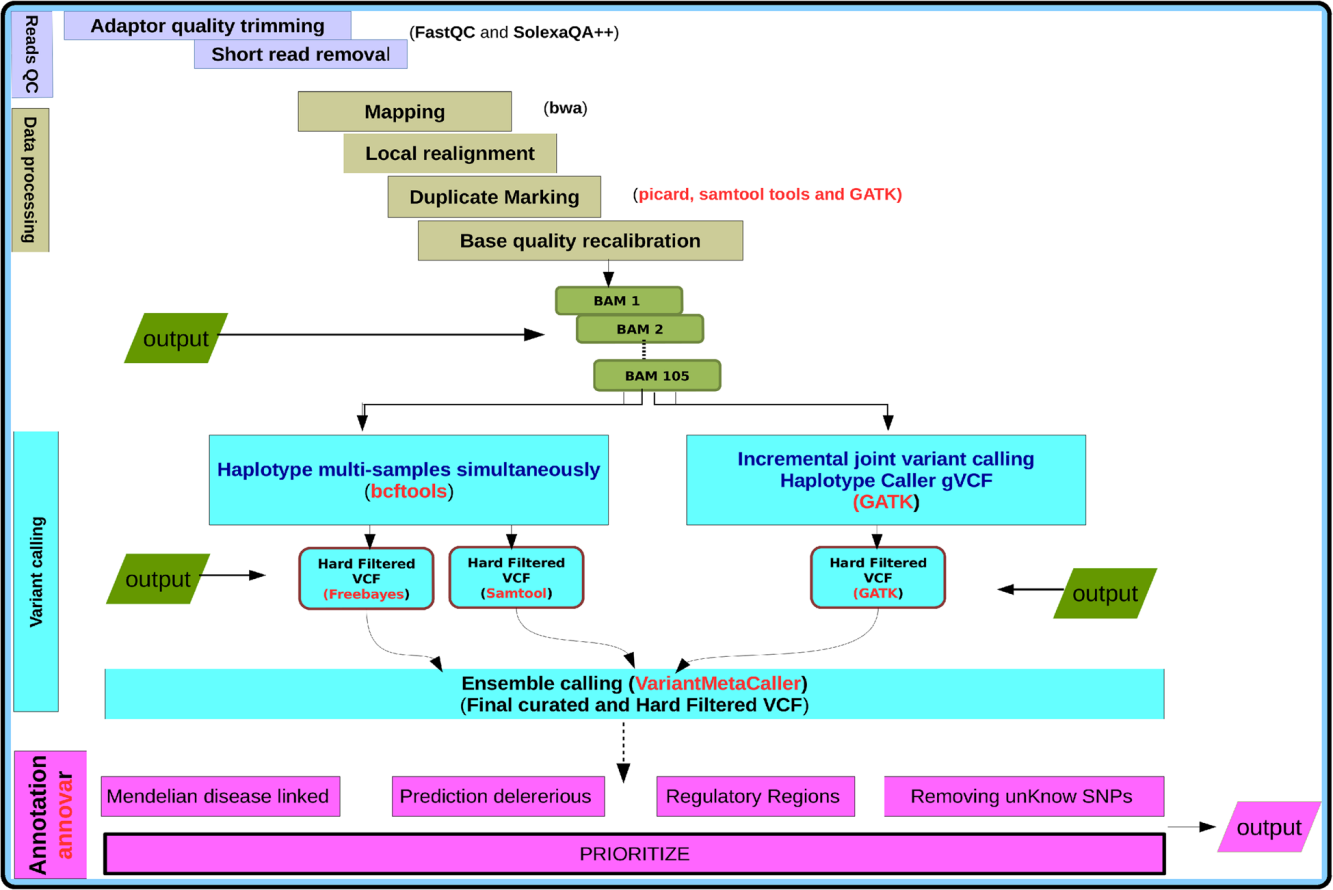
Workflow of the data analysis. Describes the bioinformatics pipelines from alignment of DNA reads, variants calling to in silico mutation prioritization

Figure 1 illustrates and summarizes the pipeline used from alignment to prioritization of mutation. We reconstructed the reads by realigning them to the complete reference genome build hg38 using BWA [25]. The Picard tool kit [26] was used to sort and mark reads duplication, after alignment. We used an ensemble approach implemented in VariantMetaCaller [27] that may find a call consensus in detecting SNPs and short indels [28]. The best practice specific to each caller were adopted [29]. We combined information generated from two independent variant caller pipelines: (1) An incremental joint variant discovery implemented in GATK 3.0 HaplotypeCaller [26], which calls samples independently to produce gVCF files and leverages the information from the independent gVCF file to produce a final call-set at the genotyping step; (2) bcftools via mpileup [30, 31] variant callers (Fig. 1). The final call-set from each subject group was produced from VariantMetaCaller [27].

### Annotation, in silico prediction of mutation and prioritization

High confidence variants were called using VariantMetaCaller [27] from the dataset including 14 Tanzanian SCD patients (nine with high HbF and five with low HbF levels). We used ANNOVAR [32] to perform gene-based annotation to detect whether SNPs cause protein coding changes and to produce a list of the amino acids that are affected. ANNOVAR contains up to 21 different functional scores including SIFT [33, 34], LRT [35], MutationTaster, MutationAssessor [36], FATHMM [37], fathmm-MKL [38], RadialSVM, LR, PROVEAN, MetaSVM, MetaLR, DANN, M-CAP, Eigen, GenoCanyon [39], CADD [40], GERP++ [41], Polyphen2 HVAR, Polyphen2 HDIV [42] and PhyloP, SiPhy [43].

From the resulting functional annotated dataset, we first filtered variants for rarity, exonic variants, non-synonymous, stop codons, predicted functional significance and deleteriousness [33, 34]. First, the resulting functional annotated data set was independently filtered for predicted functional status (of which each predicted functional status is “deleterious” (D), “probably damaging” (D), “disease_causing_automatic” (A) or “disease_causing” (D) [44–46] from these 21 in silico prediction mutation tools. Recent evaluation of in silico prediction tools for mutation effects suggested these tools are quite similar [47]. However, the evaluation of these tools was conducted mostly in non-African populations. Here we opted for an extreme casting vote approach to retain only a variant if it had at least 17 predicted functional status “D” or “A” out of 21, as one can expect a true in silico mutant variant to similarly be reported from most of these tools. Second, the retained variants were further filtered for rarity, exonic variants, nonsynonymous mutations, yielding a final candidate list of predicted mutant and genetic modifier variants.

### Network and enrichment analysis

To find out how predicted in silico mutant and modifier genes interact with others at the systems level, we analyzed how the set of all interactive genes from knowledge-based Protein-Protein Interaction (PPI) interacted with our identified in silico mutant genes and the rest of targeted genes, respectively. This has enabled the identification of potential biological pathways in which these genes participate. To achieve this, we first mapped the identified mutant SNPs to their closest genes. We mapped genes to a comprehensive human PPI network [48, 49] to identify sub-networks containing mutant and genetics modifier variant genes and their interactions. Using the Enrichr software [50], we examined how closely these genes within the extracted sub-networks are associated with human phenotypes and elucidate biological processes and pathways in which these genes participate, molecular functions and association with potential human phenotypes. The most significant pathway enriched for genes in the networks were selected from KEGG [51], Panther [52], Biocarta [53] and Reactome [54]. Gene ontologies, including molecular functions and biological processes, from the Gene Ontology database [55].

## Results

### Sample characterization

This study involved 14 SCD individuals with extreme (9 with high and 5 with low) HbF levels. Table 1, describes the age and HbF ranges of the included individuals.

**Table 1:** Characteristics of Tanzanian individuals sickle cell disease (SCD) with extreme fetal hemoglobin levels

	High HbF ≥ 7.7%	Low HbF ≤ 2.5%
N	9	5
Age range (Years)	5–19	8–21
HbF (%)	15–32	0.3–2.2

### Summary of variants found in individuals with high and low HbF levels

A total of 873 and 1196 highly confident variants were determined in SCD patients with high and low HbF levels, respectively, on chromosomes 2, 6, 7, 11, 12 and 19. Surprisingly, this shows a difference in the overall variation between the two groups of individuals with SCD.

**Fig. 2:**
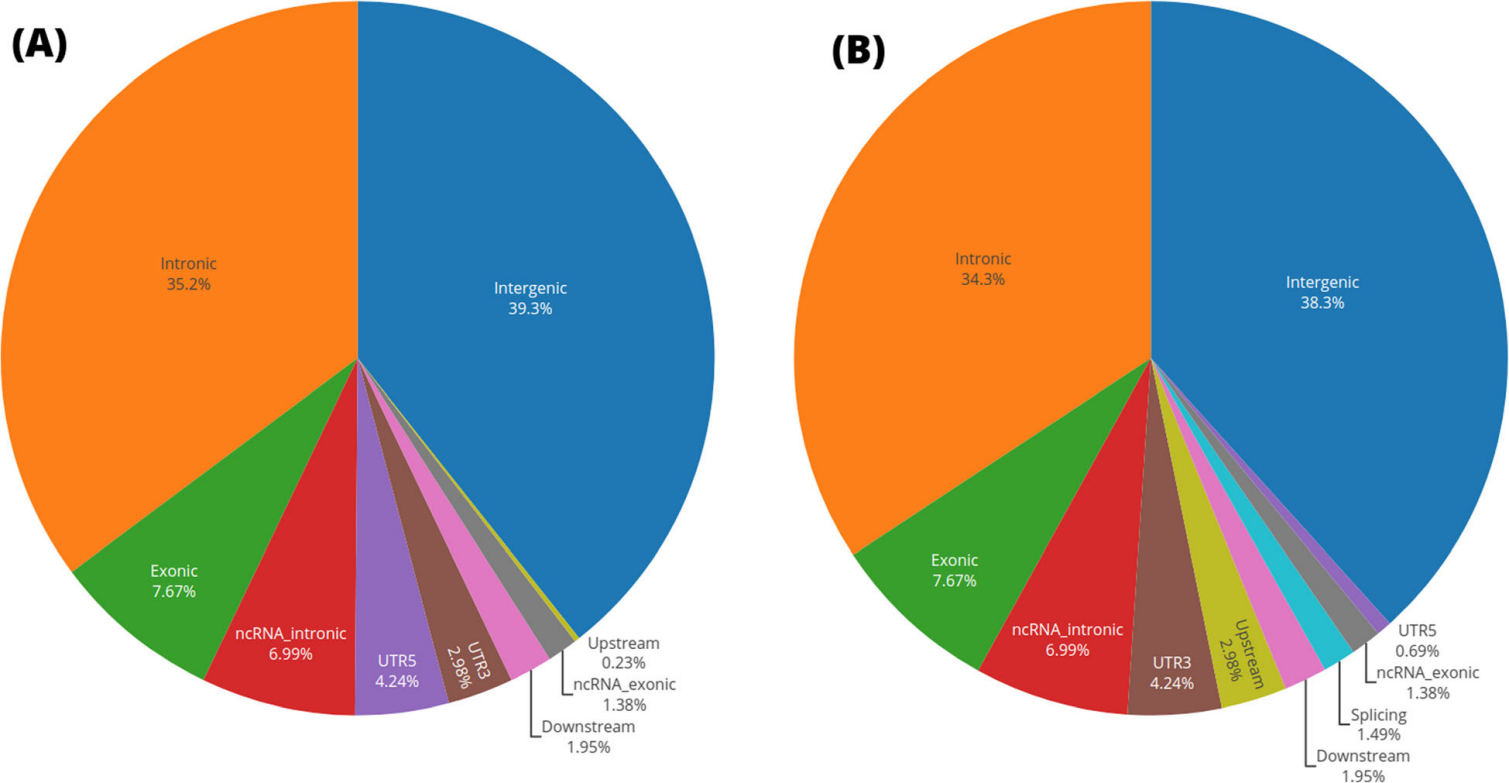
Characterization of SCD gene function and exome map from the targeted next generation sequencing: This included the exon and full regions for validated and novel fetal hemoglobin-associated loci, including *B-cell lymphoma/leukemia 11A* (*BCL11A*), *proto-oncogene, transcription factor* (*MYB*), *Homeobox A9* (*HOXA9*), *Hemoglobin subunit beta* (*HBB), hemoglobin subunit gamma 1* (*HBG1*), *hemoglobin subunit gamma 2* (*HBG2*), *chromodomain helicase DNA binding protein 4* (*CHD4*), *Kruppel like factor 1* (*KLF1*), *methyl-CpG binding domain protein 3* (*MBD3*), *zinc finger and BTB domain containing 7A* (*ZBTB7A*), *Peptidoglycan recognition protein 1* (*PGLYRP1*) on chromosomes 2, 6, 7, 11, 12 and 19, respectively. **a** gene functions from patients with high HbF levels and **b** gene functions from patients with low HbF levels

The identified variants are comprised of 77 and 82% biallelic SNPs, 0.15 and 0.11% multi-allelic SNPs, 11 and 0.9% deletions and 0.9 and 0.7% insertions in patients with high and low HbF levels (adjusted χ2 *p*-values = 1.16e-03 and 2.96e-06, as compared to uniform distribution), respectively. From these discovered variants, we detect 1 and 0 frameshift-deletions, 2 and 4 frameshift-insertions, 1 and 1 non-frameshift-insertions, 34 and 41 nonsynonymous, 3 and 3 stop-gain, 49 and 60 synonymous variants in SCD individuals with high/low HbF level, respectively. Based on our targeted chromosomal sequencing, we found significant difference in coverage of variants in the molecular structure (Fig. 2) between SCD patients with high and low HbF level (adjusted Fisher exact *p*-value = 6.1e-04), at 3’untranslated region (3’UTR) (2.98% versus 4.24%), 5′ untranslated region (5’UTR) (4.24% versus 0.69%), upstream (0.23, 2.98%). Critically, we observed that patients with high HbF have 0% variants in splicing regions, while patients with low HbF level have 1.49% (Fig. 2).

### Potential pathogenic variants

Because African-specific reported pathogenic variants are underrepresented in current databases of pathogenic variants [56], here we aimed at descriptively characterizing possible pathogenic variants from the set of polymorphisms in the retained candidate in silico mutant genes and our initial target genes discovery variants between the two patient groups. Following our pipeline and mutation prioritization, we identified six SNPs in genes (*ZBTB7A, CHD*4, *HBB, PGLYRP1, MBD3* and *MYB*) with functional impact (Table 2 and Supplementary File: Table S2) in both data generated from the SCD patients with high and low HbF levels. Two genes, *CHD4* and the *MBD3,* were found with a difference in the number of pathogenic variants (Table 3 and Supplementary File: Table S2): individuals with SCD with low HbF levels were found to have more pathogenic, benign or uncertain significant pathogenic variants.

**Table 2:** Characterization of polymorphisms within mutant and modifiers genes in SCA patients from Tanzania. Details of gene variants can be found in Supplementary File: Table S1

(High; low HbF level)								
Gene	#Polymorphisms	#MNP	#SNPs	#Deletion	#Insertion	#Pathogenic	#Benign	#USig^*^
***PGLYRP1***	**4; 4**	**0; 0**	**4; 4**	**0; 0**	**0; 0**	**0; 0**	**1; 0**	**3;4**
***ZBTB7A***	**13; 11**	**0; 1**	**10; 9**	**0; 0**	**1; 1**	**0; 0**	**2; 2**	**11;9**
***CHD4***	**25; 32**	**3; 3**	**19; 27**	**1; 0**	**1; 3**	**2; 5**	**3; 4**	**20;23**
***MBD3***	**14; 19**	**0; 2**	**12; 14**	**1; 1**	**1; 4**	**1; 2**	**0; 1**	**12;17**
***KLF1***	**11; 4**	**1; 0**	**10; 4**	**0; 0**	**0; 0**	**0; 0**	**1; 1**	**10;3**
***MYB***	**24; 27**	**1; 1**	**20; 23**	**0; 2**	**3; 1**	**0; 0**	**3; 1**	**21;26**
***BCL11A***	**27; 27**	**1; 2**	**25; 21**	**1; 2**	**0; 2**	**0; 0**	**4; 1**	**23; 26**
***HBG2***	**5; 17**	**1; 1**	**3; 12**	**0; 2**	**1; 2**	**0; 0**	**0; 0**	**5; 17**
***HOXA9***	**2; 2**	**0; 0**	**1; 1**	**0; 0**	**1; 1**	**0; 0**	**0; 0**	**2; 2**
***HBB***	**9; 10**	**0; 0**	**9; 10**	**0; 0**	**0; 0**	**0; 0**	**1; 1**	**8; 9**

*Abbreviation*: USig^*^ is the number variant with uncertain significance of pathogenicity

**Table 3:** Genes with high deleterious and loss-of-function mutations in SCA patients from Tanzania. Details of mutation on SNPs below can be found in Supplementary File: Table S2

CHR	Gene	#SNPs (High; low HbF level)	Exonic Function	# SP^**1**^
chr19	*ZBTB7A*	2; 1	Nonsynonymous	MutationTaster, FATHMM, fathmm-MKL, RadialSVM, LR, PROVEAN, MetaSVM, MetaLR, CADD, GERP++, DANN, M-CAP, Eigen, GenoCanyon, Polyphen2 HVAR, Polyphen2 HDIV, PhyloP and SiPhy
chr12	*CHD4*	11; 4	Nonsynonymous	SIFT, LRT, MutationTaster, MutationAssessor, FATHMM, fathmm-MKL, RadialSVM, LR, PROVEAN, MetaSVM, MetaLR, CADD, GERP++, DANN, M-CAP, GenoCanyon, Polyphen2 HVAR, Polyphen2 HDIV
chr11	*HBB*	3; 2	Nonsynonymous	SIFT, LRT, MutationAssessor, FATHMM, fathmm-MKL, RadialSVM, LR, ROVEAN, MetaSVM, MetaLR, CADD, DANN, Polyphen2 HVAR, Polyphen2 HDIV, PhyloP and SiPhy
chr19	*PGLYRP1*	4; 4	Nonsynonymous	SIFT, LRT, MutationAssessor, FATHMM, fathmm-MKL, RadialSVM, LR, PROVEAN, MetaSVM, DANN, M-CAP, GenoCanyon, Polyphen2 HVAR, Polyphen2 HDIV, PhyloP and SiPhy
chr19	*MBD3*	1; 2	Stop-gain	SIFT, LRT, MutationTaster, MutationAssessor, LR, PROVEAN, MetaSVM, MetaLR, CADD, GERP++, DANN, M-CAP, Eigen, GenoCanyon, Polyphen2 HVAR, Polyphen2 HDIV, PhyloP and SiPhy
chr6	*MYB*	1; 1	Nonsynonymous	SIFT, LRT, MutationTaster, MutationAssessor, FATHMM, fathmm-MKL, RadialSVM, LR, PROVEAN, MetaSVM, MetaLR, CADD, GERP++, DANN, M-CAP, GenoCanyon, Polyphen2 HVAR, Polyphen2 HDIV, PhyloP

*Abbreviation*: *# SP*^*1*^ is the number of in silico mutation tools predicted and considered damaging

Individuals with SCD with lower HbF levels had a significantly higher number of variants with insertions at both *CHD4* and *MBD3* than patients with high HbF levels (Table 2 and Supplementary File: Table S1). While both groups have small numbers of deletion variants, individuals with low HbF level had fewer deletions than those with high HbF level.

Based on Exome Aggregation Consortium (ExAC) database of pathogenic mutation [25], we found no significant difference in the number of pathogenic variants in both SCD patients with high or low HbF levels in genes (*BCL11A*), proto-oncogene, *transcription factor* (*MYB*), *Homeobox A9* (*HOXA9*), *hemoglobin subunit gamma 2* (*HBG2*), *Kruppel like factor 1* (*KLF1*), *zinc finger and BTB domain containing 7A* (*ZBTB7A*) in chromosomes 2, 6, 7, 11, 12 and 19, respectively. Overall, our targeted next generation sequencing of HbF associated genetic loci identified a disproportional number of loci with a few variants, particularly deletions, present in patients with high levels of HbF.

### Biological pathways and processes associated with genes with high mutational burdens

**Fig. 3:**
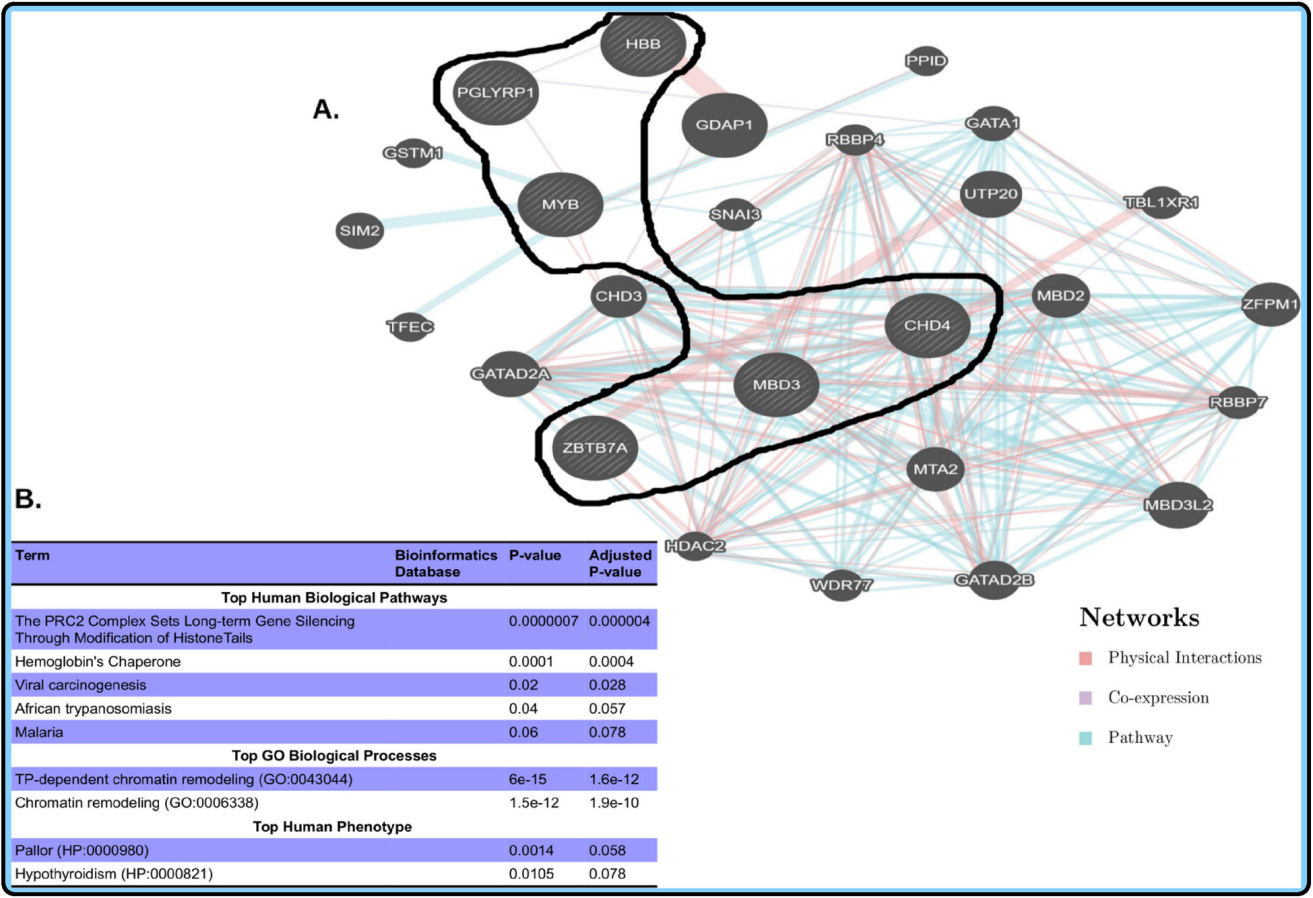
Biological sub-network of the candidate mutant gene and identified modifier genes in 14 SCD patients from Tanzania. **a** sub-networks of the mutant gene and identified candidate genetic modifiers include *CHD4 and MBD3*. **b** description of the top most significant pathways, GO biological process, and Human Phenotypes associated with the identified variants

**Fig. 4:**
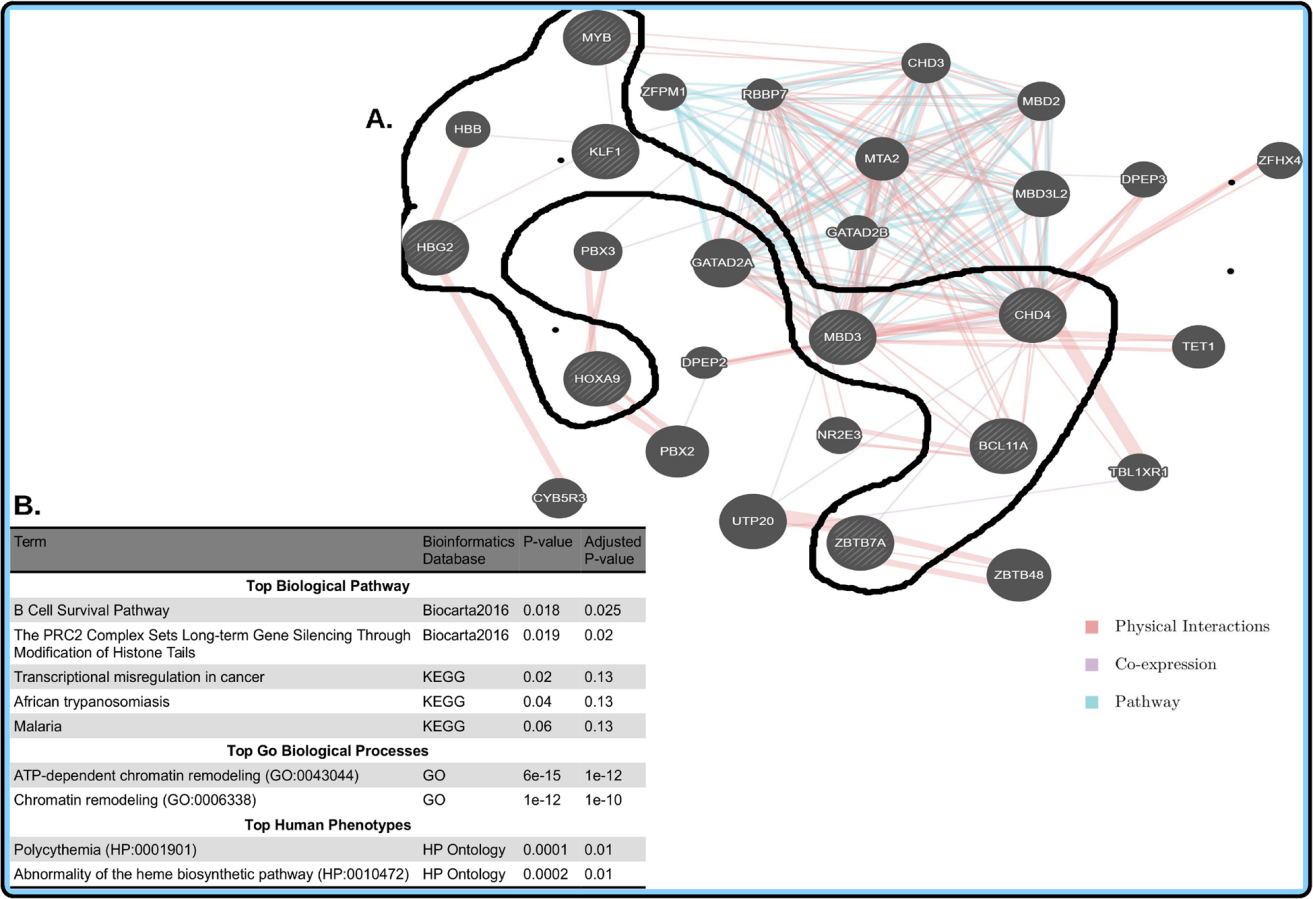
Biological sub-network of the candidate mutant gene and identified modifier genes in 14 SCD patients from Tanzania. **a** sub-networks of our target sequencing variants include *ZBTB7A, BCL11A, MYB, HBB, HOXA9, HBG2, CHD4, KLF1, MBD3.***b** description of the top most significant pathways, GO biological process, and Human Phenotypes associated with the identified variants

Independent roles of the identified candidate in silico mutant genes (Supplementary File: Table S1, Fig. 2) or our initial targeted nine genes are known in Sickle Cell disease. However, how these genes interact with others at the systems level is currently unknown in various populations of African SCD patients. As described in the Methods section, using the set of all interactive genes including our identified mutant genes and the rest of targeted genes may contribute in identifying potential Sickle Cell-specific pathways in which modifier and mutant genes participate together in conferring variation in Sickle Cell Disease severity. The identified Protein-Protein Interaction (PPI) sub-network formed from 2 genes (Fig. 3a) showed an enrichment of rare variants with deleterious effects was enriched for the *PRC2 complex* which influence long-term gene silencing through modification of histone tails (*P* = 0.000004; Fig. 3b), and is highly associated with or involved in the *TP-dependent chromatin remodeling* (*P* = 1.6e-12, Fig. 3b) biological process, nominally associated with *pallor* (*P* = 0.0014, Fig. 3b). *CHD4* and *MBD3* were found to be the most important genes (hubs) of sub-network (Fig. 4a), which are nominally associated with the B cell survival pathway (*P* = 0.018, Fig. 4b), known to be implicated in the *ATP-dependent chromatin re-modeling* biological process (*P* = 6e-15, Fig. 4b) and associated with *polycythemia disorder* (*P* = 0.0001, Fig. 4b).

## Discussion

This is the first study in Africa to conduct targeted next generation sequencing to investigate genetic modifiers and pathways associated with extreme fetal hemoglobin (HbF) in individuals with SCD. Most of the loci (SNPs) that have been found to associate with HbF by GWAS only show possible associations with variants covered by the array chip used. The approach taken in this study was to perform in-depth sequencing around previously identified loci to descriptively compare, in detail, discovered polymorphisms between individuals with extreme HbF levels. We have identified single nucleotide polymorphisms (SNPs), insertions (IN) and deletions (DEL) across 8 targeted regions in chromosomes 2, 6, 7, 11, 12 and 19. We found differing types of polymorphisms, including SNPs and INDELS between individuals with low HbF versus those with high HbF, suggesting potential modifier effect. Interestingly, key discovered variants, together with previously identified variants, are enriched in biological pathways that underlie the HbF regulation.

It is worth also noting that possible structural variants in these patient groups may make the sequencing off between the two groups. Furthermore, current challenges, including (1) limitation of variant calling tools in African data [57], (2) sequencing errors and structural variants in African data [58] and (3) under-representation of African samples in the current reference genome [58, 59], may contribute to the observed difference in variants discovered in both high and low HbF level in individuals with SCD. We found more deletions in individuals with high HbF than those with low HbF levels indicating their role in HbF synthesis pathways. A number of significant deletions have been reported before, particularly in the globin cluster [60–62]. In this study, we have identified additional potential deletions across the targeted regions (Table 2). We observed more insertions in individuals with high HbF than in those with low HbF. However, frameshift deletions were more prevalent in individuals with high HbF, while frameshift insertions were more prevalent in individuals with low HbF. Frameshift deletions and insertion may lead to abnormal proteins due to shorter or longer sequences, respectively.

We also looked at variants located at untranslated regions (UTR) both at 3′ and 5′ ends which are involved differently in regulation of gene expression. Interestingly, in individuals with high HbF levels, variants in the 5’UTR were more prevalent as opposed to more variants in the 3’UTR in individuals with low HbF levels. Molecular mechanisms of the 5’UTR include regulating translation of main coding sequences while the 3’UTR contain binding sites for microRNA (miRNA) which takes part in the timing and rate of translation of the corresponding mRNA. Hence the difference in variants in these two regions between individuals with high HbF versus those with low HbF is notable and may contribute differently in the regulation of HbF synthesis.

We looked specifically at non-synonymous mutations and found that out of the eight targets, six were found to have mutations with functional impact. Of interest, the genes *CHD4* and *MBD3*, functionally interacting in the same sub-network (see Fig. 3), had more pathogenic mutations in individuals with low HbF levels than those with high HbF. *CHD4* is a chromatin organization modifier which confers the chromatin remodeling function of the NuRD complex. *CHD4* has been reported to repress *γ-globin* gene expression in mice [63, 64]. Similarly, *MBD3* operates as a NuRD complex and is associated with the transcription factors GATA-1 and FOG-1, which directly regulate genes within the β-globin locus.

The human protein-protein interaction (PPI) (Fig. 3a) for CHD4 and MBD3 proteins indicates that they are essential to system survival and hence their biological functions tend to be evolutionary conserved [63]. Thus, in presence of non-synonymous mutations, it is expected that individual components (proteins and interactions) in the system must adapt to a changing environment while maintaining the system’s primary function. In this study, we observed that, to maintain its robustness while sustaining its function under fluctuating environmental conditions, the system possibly triggers different mechanisms. This ensures that the network retains the modularity degree in order to provide a selective advantage for the host system by conserving and/or gaining useful functional interactions within the network to ensure an increase of HbF levels. As an illustration, *CHD4*, as well as *MBD3*, indirectly interact with *KLF1* and *MYB*, which are potent activators of *BCL11A*. *CHD4* is believed to exert its gamma globin silencing effect by positively regulating the *BCL11A* and *KLF1* genes. In addition, *BCL11A* and *MYB* are known to be involved in γ-globin gene regulation, leading to either elevation or reduction of HbF levels [64]. The difference in frequency of non-synonymous mutations in the individuals with high HbF levels versus those with low levels reflect different interactions within this network and the resulting levels of HbF.

Though these post-analysis results are consistent with the literature and are biologically relevant, it is worth noting that, due to relatively high noise related to high-throughput data or experiments from which interactions are inferred, the protein-protein interaction network used may contain incorrectly classified interactions, i.e., failing to detect interactions (false negatives) or wrongly identifying some other interactions (false positives). This suggests these results still need to be validated experimentally. In this study, we minimized the likelihood of incorrectly classified interaction computationally by: (1) using a data integration model, combining information from multiple interacting data sources into one unified network, and (2) applying a strict interaction reliability or confidence score cutoff. These techniques are expected to significantly reduce the false negative and positive rate of the network produced, leading to a PPI network of high confidence interactions with an increased coverage [65].

Given our study design, we did not perform genetics differentiation tests or statistical tests of differences in minor allele frequencies or genotype counts. Instead, we have aimed at descriptively characterizing the proportion of variants between the low/high HbF from high confident variants calling, compare the count of pathogenic variants between the groups and identify potential Sickle Cell-specific pathways in which modifier and mutant genes participate in conferring variation in Sickle Cell severity. Importantly, our current study suggests (1) a difference in the overall genetic variation between Sickle Cell patients with high and low HbF level and, (2) biological pathways, including the *PRC2 complex* which sets long-term gene silencing through modification of histone tails (*P* = 0.000004; Fig. 3b), hemoglobin’s Chaperone (*P* = 0.001, Fig. 4b) and B-cell Survival (*P* = 0.018, Fig. 4b). These identified pathways may harbour potential interactive Sickle Cell-specific genes including modifier, mutant and other genes (Figs. 3 and 4) in conferring variation in severity among individuals with SCD. This work has focused on the importance of studying both genetic and epigenetic pathways in HbF regulation. Our findings suggest an in-depth whole genome sequencing study to fully characterize modifier genes implicated in the variation of SCD severity. This approach may contribute to future development of interventions for SCD, including drugs and gene therapy. Finally, to note is, the modest sample size limited the expected statistical power, which could yield false positive associations and missed others. However, different results obtained provide a strong hypothesis for future studies. With a larger sample size, it would be possible to perform genetics differentiation tests or statistical tests of differences in minor allele frequencies or genotype counts and possibly identify additional essential variants and biological pathways associated with extreme HbF levels in SCD using the model set by this study.

## Conclusions

This study has shown that the analysis of genetic modifiers associated with HbF in SCD patients can elucidate genetic factors underlying extreme (low or high) HbF levels in these patients. The study has identified frameshift deletion in SCD patients with high HbF levels and frameshift insertions in both *CHD4* and *MBD3* for those with low HbF, and some of these insertions are associated with the SCD pathogenesis.

## Supplementary information

**Additional file 1: Table S1.** Summary of chromosomal positions and sequenced regions of the targeted genes.

**Additional file 2: Table S2.** Details of gene variants identified in SCD patients from Tanzania.

## Data Availability

Additional supporting information can be found in the supplementary file at the end of the article. The datasets used are available at the European Genome-phenome Archive (EGA), accession number EGAS00001000990, and accessible via the following link: https://www.ebi.ac.uk/ega/studies/EGAS00001000990

## Abbreviations

ANNOVAR: ANNOtate VARiation
BCF: Binary variant call format
BCL11A: B-cell lymphoma/leukemia 11A
BWA: Burrows-Wheeler Alignment
CHD4: Chromodomain helicase DNA binding protein 4
DNMT1: DNA methyltransferase 1
ExAC: Exome Aggregation Consortium
FATHMM: Functional Analysis through Hidden Markov Models
FOG1: Friend of GATA1
GATA1: GATA Binding Protein 1
GVCF: Genomic variant call format
GWAS: Genome Wide Association Study
HbA: Hemoglobin A
HbAs: Hemoglobin AS
HBB: Hemoglobin Subunit beta
HbF: Fetal hemoglobin
HBG2: Hemoglobin subunit gamma 2
HbS: Hemoglobin S
HOXA9: Homeobox A9
INDELS: Insertions and deletions
KCRI: Kilimanjaro Clinical Research Institute
KLF1: Kruppel like factor 1
LSD1: Lysine-specific demethylase 1
MBD3: Methyl-CpG binding domain protein 3
MYB: Myeloblastosis transcription factor
NuRD: Nucleosome remodeling and deacetylase
PPI: Protein–protein interaction
PRC2: Polycomb repressive complex 2
SCD: Sickle cell disease
SNP: Single nucleotide polymorphisms
TR2: Testicular nuclear receptor 2
TR4: Testicular nuclear receptor 4
UTR: Untranslated region
ZBTB7A: Zinc finger and BTB domain containing 7A
