# Supplementary material for: Identifying genetic variants and pathways associated with extreme levels of fetal hemoglobin in sickle cell disease in Tanzania
Source: BMC Med Genet. 2020 Jun 5;21:125. doi: 10.1186/s12881-020-01059-1 (PMC7275552; doi:10.1186/s12881-020-01059-1)
Supplement: Supplementary file 1 — Additional file 1: Table S1. Summary of chromosomal positions and sequenced regions of the targeted genes. [file 12881_2020_1059_MOESM1_ESM.docx]

**Table S1:** Summary of chromosomal positions and sequenced regions of the targeted genes

| **Target genes** | **Target Region Name** | **Chr.** | **Start Pos.** | **End Pos.** |
| --- | --- | --- | --- | --- |
| BCL11A | BCL11A Merged_UserDefined (55884792) | 2 | 60684324 | 60689564 |
| BCL11A | BCL11A_Exon (54487059) | 2 | 60773106 | 60773435 |
| BCL11A | BCL11A_Exon (54487062) | 2 | 60695867 | 60695968 |
| BCL11A | BCL11A_FiveUtrExon (54487063) | 2 | 60780351 | 60780633 |
| BCL11A | BCL11A_ThreeUtrExon (54487060) | 2 | 60678302 | 60679801 |
| MYB | MYB Merged_UserDefined (55851211) | 6 | 135515489 | 135515603 |
| MYB | MYB Merged_UserDefined (55851212) | 6 | 135516881 | 135517145 |
| MYB | MYB Merged_UserDefined (55851213) | 6 | 135521218 | 135521558 |
| MYB | MYB Merged_UserDefined (55884804) | 6 | 135510924 | 135511490 |
| MYB | MYB Merged_UserDefined (55884805) | 6 | 135538997 | 135540316 |
| MYB | MYB_Exon (54487070) | 6 | 135508972 | 135509043 |
| MYB | MYB_Exon (54487072) | 6 | 135522777 | 135522887 |
| MYB | MYB_Exon (54487075) | 6 | 135520046 | 135520188 |
| MYB | MYB_Exon (54487079) | 6 | 135513462 | 135513696 |
| MYB | MYB_Exon (54487080) | 6 | 135507041 | 135507158 |
| MYB | MYB_Exon (54487081) | 6 | 135518099 | 135518461 |
| MYB | MYB_Exon (54487082) | 6 | 135524355 | 135524462 |
| MYB | MYB_Exon (54487083) | 6 | 135514976 | 135515056 |
| MYB | MYB_FiveUtrExon (32197136) | 6 | 135502453 | 135502674 |
| HOXA9 | HOXA9_FiveUtrExon (53660082) | 7 | 27204497 | 27205149 |
| HBG1 | Coordinate_UserDefined (55884716) | 11 | 5269202 | 5271087 |
| HBG2 | Coordinate_UserDefined (55884718) | 11 | 5274121 | 5276395 |
| HBB | Coordinate_UserDefined (55884720) | 11 | 5246396 | 5248301 |
| CHD4 | CHD4 Merged_UserDefined (55884793) | 12 | 6679243 | 6680203 |
| CHD4 | CHD4 Merged_UserDefined (55884794) | 12 | 6686946 | 6687717 |
| CHD4 | CHD4 Merged_UserDefined (55884795) | 12 | 6690205 | 6690985 |
| CHD4 | CHD4 Merged_UserDefined (55884796) | 12 | 6691776 | 6692549 |
| CHD4 | CHD4 Merged_UserDefined (55884797) | 12 | 6696545 | 6697120 |
| CHD4 | CHD4 Merged_UserDefined (55884798) | 12 | 6700627 | 6701228 |
| CHD4 | CHD4 Merged_UserDefined (55884799) | 12 | 6701554 | 6702787 |
| CHD4 | CHD4 Merged_UserDefined (55884800) | 12 | 6707055 | 6707596 |
| CHD4 | CHD4 Merged_UserDefined (55884801) | 12 | 6708934 | 6711668 |
| CHD4 | CHD4_Exon (54487091) | 12 | 6691303 | 6691447 |
| CHD4 | CHD4_Exon (54487100) | 12 | 6682240 | 6682435 |
| CHD4 | CHD4_Exon (54487104) | 12 | 6697464 | 6697588 |
| CHD4 | CHD4_Exon (54487111) | 12 | 6705172 | 6705303 |
| CHD4 | CHD4_Exon (54487114) | 12 | 6704500 | 6704596 |
| CHD4 | CHD4_Exon (54487115) | 12 | 6688012 | 6688083 |
| CHD4 | CHD4_Exon (54487120) | 12 | 6703625 | 6703816 |
| CHD4 | CHD4_FiveUtrExon (54487116) | 12 | 6716466 | 6716551 |
| CHD4 | CHD4_FiveUtrExon (54487117) | 12 | 6715440 | 6715617 |
| MBD3 | MBD3_Exon (54487065) | 19 | 1582621 | 1582711 |
| MBD3 | MBD3_Exon (54487066) | 19 | 1585054 | 1585213 |
| MBD3 | MBD3_Exon (54487067) | 19 | 1581091 | 1581268 |
| MBD3 | MBD3_Exon (54487068) | 19 | 1584539 | 1584676 |
| MBD3 | MBD3_FiveUtrExon (54487069) | 19 | 1592521 | 1592652 |
| MBD3 | MBD3_ThreeUtrExon (54487064) | 19 | 1576678 | 1578537 |
| ZBTB7A | ZBTB7A_FiveUtrExon (54487054) | 19 | 4053969 | 4055245 |
| ZBTB7A | ZBTB7A_FiveUtrExon (54487055) | 19 | 4066680 | 4066816 |
| ZBTB7A | ZBTB7A_ThreeUtrExon (54487056) | 19 | 4045216 | 4048242 |
| KLF1 | KLF1 Merged_UserDefined (55884802) | 19 | 12995232 | 12996961 |
| KLF1 | KLF1_FiveUtrExon (54487050) | 19 | 12997868 | 12998017 |
| PGLYRP1 | Coordinate Merged_UserDefined (55884803) | 19 | 46522392 | 46522925 |
| PGLYRP1 | Coordinate_FiveUtrExon (54487133) | 19 | 46525993 | 46526556 |

Genome build: hg19
